# Scoping Review—The Effectiveness of Clear Aligners in the Management of Anterior Open Bite in Adult Patients

**DOI:** 10.3390/medicina61061113

**Published:** 2025-06-19

**Authors:** Nicolae Daniel Olteanu, Cristian Romanec, Eduard Radu Cernei, Nikolaos Karvelas, Livia Nastri, Irina Nicoleta Zetu

**Affiliations:** 1 Department of Oral and Maxillofacial Surgery, Faculty of Dental Medicine, “Grigore T. Popa” University of Medicine and Pharmacy from Iasi, 16 Universitatii Str., 700115 Iasi, Romania; daniel.olteanu@umfiasi.ro (N.D.O.); liviu.romanec@umfiasi.ro (C.R.); karvelas93@gmail.com (N.K.); irina.zetu@umfiasi.ro (I.N.Z.); 2 Multidisciplinary Department of Medical-Surgical and Dental Specialties, University of Campania “Luigi Vanvitelli”, 80138 Naples, Italy; livia.nastri@unicampania.it

**Keywords:** clear aligner, Invisalign^®^, anterior open bite, anterior extrusion, posterior intrusion, malocclusion, apertognathia, nonocclusion

## Abstract

*Background and Objectives*: Anterior open-bite malocclusion remains a challenging orthodontic condition where achieving a positive overbite necessitates precise control of incisor extrusion and molar intrusion. With recent advances in clear aligner therapy—improved materials, attachment techniques and digital treatment planning—the potential for non-invasive treatment has increased. This scoping review systematically maps the evidence on the efficacy of clear aligners in treating anterior open bite among adult patients, outlines treatment protocols and highlights gaps in the literature. *Materials and Methods*: A systematic search was conducted in PubMed/Medline, Embase/ScienceDirect and Clarivate/Web of Science for literature published in English between January 2000 and December 2024. Studies involving adult patients treated with clear aligners (predominantly Invisalign^®^) were included. A two-step screening process was applied, and data were charted according to pre-specified criteria. The review adheres to the PRISMA-ScR checklist guidelines. *Results*: From an initial pool of 802 articles, 30 met the inclusion criteria following duplicate removal and full-text screening. The evidence suggests that clear aligners can achieve measurable incisor extrusion and posterior intrusion when appropriate auxiliary techniques (e.g., attachments and mini screws) are used. However, digital treatment planning software may overestimate movement predictions, necessitating iterative refinement phases. Patient compliance, clinician expertise and technological limitations are key factors influencing outcomes. *Conclusions*: Clear aligner therapy represents a promising alternative to fixed appliances for anterior open-bite correction in adults, although challenges remain in achieving precise vertical control. Further high-quality randomized controlled trials and standardized outcome measures are needed to confirm long-term stability and efficacy.

## 1. Introduction

The orthodontic treatment of anterior open bite aims to guide the extrusion of the upper and lower incisors and the molar intrusion to achieve a positive overbite. The lower molar intrusion leads to counterclockwise mandibular rotation. Treatment outcomes might cause esthetic drawbacks of facial profile and smile due to the persistence of large mandibular and occlusal plane angles [1]. Improvements in anterior extrusion and posterior intrusion, added in 2011, when Invisalign^®^ clear aligners were provided with additional attachments, mini-screws and mini-plates, make it worth reconsidering the earlier reluctance to use aligners for open-bite treatment [2]. A meta-analysis on the long-term stability of anterior open-bite treatment, with the last literature search performed in April 2009, reported solely fixed orthodontic appliances as a means of nonsurgical treatment [3]. However, a later review, based on a search up to April 2019, found that clear aligners (CAs) can be used in anterior open-bite cases caused by the intrusion of anterior teeth [4].

Open-bite malocclusions of transversal, sagittal or vertical cause can be treated with CAs. The decision for posterior intrusion or upper incisor extrusion is made following a smile analysis and the evaluation of the facial lower-third type. Attachments are required for the anterior extrusion. Open bites are classified as mild (up to −2 mm), moderate (−3 mm to −4 mm) and severe (higher than −4 mm). In easy open-bite cases treated with a CA, no posterior intrusion is required, whereas a posterior intrusion of less than 1 mm should be achieved in moderate cases, and auxiliary techniques are necessary in complex open-bite cases [5].

An updated scoping review of the evidence in the literature on clear aligner efficacy in open-bite treatment in adult patients could provide useful information in a rapidly evolving field. By identifying the benefits of CAs other than esthetic ones in the most difficult to correct malocclusions, more patients and dentists could be encouraged to abandon braces in favor of CAs, which have the advantage of less chair time, minimal adverse effects on gingival health and the possibility of carrying out the entire procedure digitally. In the opinion of some professionals, the disadvantage of CAs is that they still have significant limitations in the treatment of complex malocclusions [2,3,4]. The addition of attachments and temporary anchorage devices and more predictable digital simulations will continuously change the outcomes of open-bite treatment using CAs [5].

The primary objective of this scoping review is to map and synthesize the current evidence on the efficacy of CAs in treating anterior open-bite malocclusion in adult patients. The specific aims are the following:

- Map the literature on treatment protocols (e.g., anterior extrusion, posterior intrusion and refinement procedures) employed with CAs;
- Evaluate clinical outcomes comparing incisor and molar movements, overbite correction and mandibular rotation effects;
- Identify key influencing factors—patient compliance, clinician expertise and aligner material properties—and outline potential limitations.

## 2. Materials and Methods

### 2.1. Research Question & Protocol

The research question was formulated to strike a balance between specificity and breadth—precise enough to facilitate the identification of relevant studies yet broad enough to capture the full scope of the topic. Specifically, this review seeks to answer the following: “Are clear aligners effective in the management of anterior open bite in adult patients?”

Based on the main research question, the review protocol was developed in accordance with the PRISMA-ScR [6] (Preferred Reporting Items for Systematic Reviews and Meta-Analyses Extension for Scoping Reviews) guidelines. The PICC framework (Population—adult patients (≥18 years) with anterior open-bite malocclusion, Intervention—clear aligner therapy, Concept—effectiveness of CAs in correcting anterior open bite, Context—orthodontic treatment settings, regardless of the geographical location or practice type) was used to structure the review approach and eligibility criteria.

### 2.2. Eligibility Criteria

Inclusion Criteria:

- Population: adult patients (≥18 years) with anterior open-bite malocclusion;
- Intervention: treatment with CA;
- Study Designs: case reports/series, retrospective and prospective observational studies and randomized controlled trial studies (RCTs);
- Language: articles published in English from January 2000 to December 2024.

Exclusion Criteria:

- Studies focusing on pediatric/adolescent patients (due to differences in growth dynamics);
- Studies lacking detailed methodological descriptions concerning treatment planning and outcome evaluation;
- Narrative reviews, systematic reviews and meta-analyses.

### 2.3. Information Sources and Search Strategy

A systematic literature search was independently conducted by two reviewers, utilizing a combination of controlled vocabulary (MeSH terms) and free-text keywords. The databases searched included PubMed/Medline, Embase/ScienceDirect and Clarivate/Web of Science, covering the period from January 2000 to December 2024. The final search was conducted on 15 February 2025.

**Figure 1:**
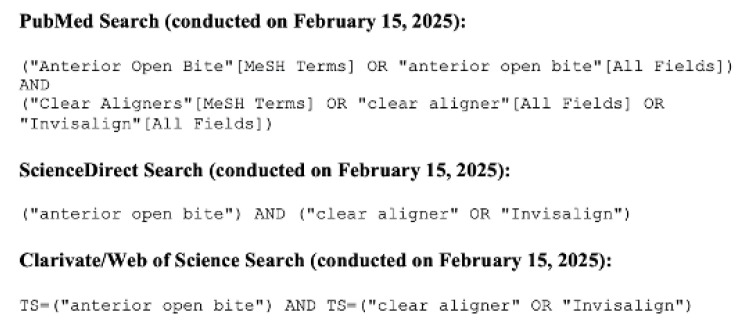
Database query details.

The search strategy combined controlled vocabulary (MeSH terms) and free-text keywords, including “anterior open bite” AND (“clear aligner” OR “Invisalign”) using Boolean operators (AND/OR) (Figure 1).

### 2.4. Selection of Sources of Evidence

Study selection was performed independently by two reviewers, following a two-stage screening process. Initially, all retrieved references were imported into Zotero 6.0.27 software, for management and deduplication. Titles and abstracts were then screened for relevance based on the predefined inclusion criteria. Articles that appeared eligible underwent full-text review to confirm inclusion. Inter-reviewer reliability was assessed during the full-text screening phase using Cohen’s kappa, yielding a value of 0.83, indicating strong agreement. Discrepancies were resolved through discussion and consensus. The final set of included studies was subjected to detailed analysis.

To ensure comprehensive coverage, additional searches were conducted in OpenGrey and Google Scholar to identify relevant gray literature. These searches yielded no additional eligible studies. All sources were screened using the same eligibility criteria as for peer-reviewed literature.

**Figure 2:**
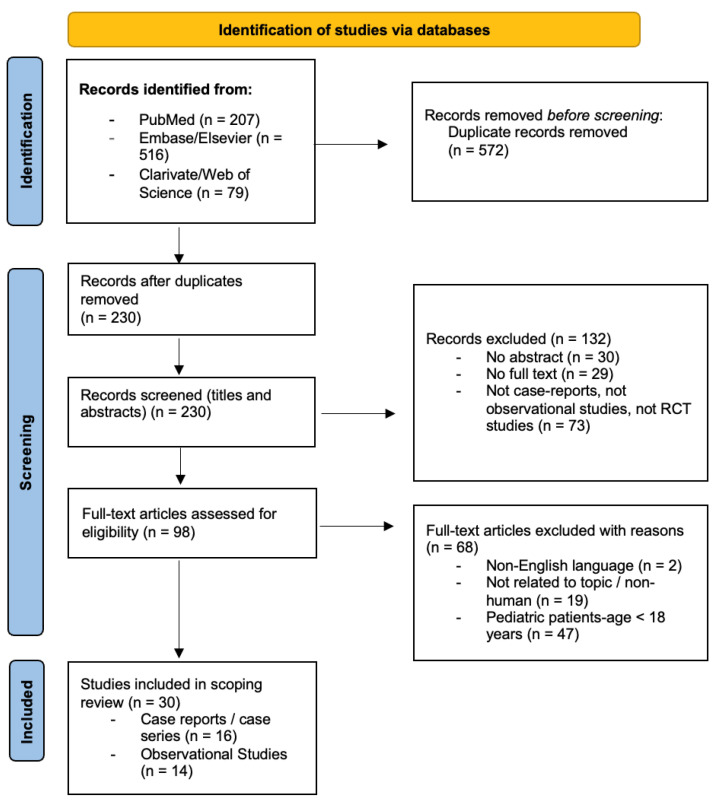
PRISMA flow diagram of the study selection.

The study selection process is outlined in the PRISMA flow diagram (Figure 2).

### 2.5. Data Charting Process

Data extraction was carried out by two independent reviewers using a pre-designed standardized data extraction form. Extracted variables included the following:

- Publication data;
- Study design, type of intervention and sample size;
- Description of the clear aligner treatment protocol (treatment phases, including anterior extrusion, posterior intrusion, refinements and auxiliary devices);
- Outcome measures: overbite correction, incisor extrusion and molar intrusion values, mandibular rotation and treatment duration;
- Reported limitations and recommendations;
- Main conclusions as reported by the study authors.

In cases where there was uncertainty or disagreement regarding data extraction, discrepancies were resolved through discussion and mutual agreement.

### 2.6. Synthesis of Results

Data were synthesized descriptively. The findings were categorized thematically to reflect the treatment protocols, clinical efficacy and influencing factors. Tables were used to visually summarize outcomes and study characteristics according to the PRISMA-ScR guidelines.

## 3. Results

### 3.1. Study Selection

The initial database search identified 802 articles. After the removal of duplicates and screening based on title/abstract and eligibility, 30 articles were included following full-text review. The study selection process is detailed in Figure 2.

### 3.2. Characteristics of Included Studies

The included literature comprised 13 case reports studies, 3 case series studies and 14 observational studies (13 retrospective studies and 1 prospective cohort study). The study sample sizes varied, and the treatment outcomes were primarily reported in terms of incisor and molar movements, overbite correction and the frequency of required refinements.

Table 1 presents a comprehensive synthesis of key biomechanical considerations, treatment refinements, retention strategies and influencing factors associated with anterior open-bite correction using CAs, supported by relevant scholarly references. Table 2 summarizes the patient parameters involved in the treatment decision making. Table 3 summarizes the clinician-related factors involved in the treatment plans. Table 4 summarizes key clinical studies evaluating the effectiveness of CAs in the correction of anterior open bite, detailing study designs, participant characteristics, biomechanical mechanisms of bite closure and measured treatment outcomes.

Sixteen studies focused on Invisalign^®^ treatment protocols, likely due to the widespread use of the ClinCheck^®^ Pro 6.0 digital treatment planning software (Table 5), whereas the remaining fourteen studies either did not specify the aligner system used or employed other clear aligner systems without detailed protocol descriptions.

Several case reports have highlighted the versatility and clinical potential of clear aligner therapy in managing anterior open-bite and complex malocclusions. Waxler (2021) [17] described the successful correction of skeletal open bite using clear aligners combined with miniscrews, emphasizing the role of skeletal anchorage in enhancing vertical control. Vadera et al. (2023) [19] reported nonsurgical management of a skeletal Class III malocclusion with dentoalveolar open bite, demonstrating the efficacy of clear aligners in addressing challenging sagittal and vertical discrepancies without surgery.

Gudhimella et al. (2022) [30] detailed the management of anterior open bite in a skeletal Class II hyperdivergent patient using clear aligner therapy, further reinforcing the appliance’s role in treating difficult vertical skeletal patterns. Greco et al. (2021) [31] reported successful anterior open-bite closure in an adult using clear aligners combined with micro-osteoperforations to enhance vertical control and treatment efficiency.

El-Bialy (2020) [27] presented a unique case combining high-frequency vibration with clear aligners to treat an adult patient with Class III skeletal malocclusion, open bite and severe bimaxillary protrusion, suggesting adjunctive therapies may optimize treatment outcomes. Tepedino et al. (2023) [33] illustrated the integration of clear aligners with myofunctional appliances to achieve anterior open-bite closure, supporting a multidisciplinary approach. Wen et al. (2022) [36] documented the clear aligner treatment of an adult with severe anterior open-bite malocclusion, highlighting the appliance’s capacity for significant vertical correction. Rodriguez (2012) [34] demonstrated a non-extraction treatment of a Class II open bite in an adult patient using clear aligners, underscoring their applicability in varied malocclusion types. Lastly, Haubrich and Schupp (2023) [35] provided a comprehensive overview of open-bite treatment with aligner orthodontics, including clinical case examples, emphasizing biomechanical considerations. Collectively, these case reports enrich the evidence base by showcasing innovative protocols, adjunctive techniques and clinical outcomes supporting the effectiveness of clear aligners in anterior open-bite correction.

## 4. Discussion

### 4.1. Treatment Goals

The treatment aims to close the dental open bite through anterior extrusion, posterior intrusion with arch expansion, flattening of the curve of Spee and, if necessary, myofunctional tongue therapy, usually in patients with tongue thrusting habits [7]. However, the upper anterior extrusion is considered unstable and detrimental to periodontal health and smile esthetics, which is why mandibular molar intrusion and mandibular incisor extrusion could be an alternative [8]. Counterclockwise rotation of the mandible is necessary for establishing an anterior overbite [37]. Open-bite treatment with aligners also causes significant incisor retraction [9]. The positive overbite of the upper and lower incisors is achieved by reducing overbite. Extractions are also used for closing the open bite with an expected protraction induced by the closure of the extraction space [8].

Some reports of solving skeletal open bite exclusively with aligners and without surgery [16] were criticized as not being sustained by the superimposition cephalometric radiographs [17,18], with the growth process being a risk of bias in a 16-year-old patient. A skeletal open-bite closure with a CA, intermaxillary elastics and composite veneers on the upper lateral incisors showed good stability after 5 years in a non-growing patient aged 19 [26]. A more recent case report of a 35-year-old patient with hypodivergent facial type, skeletal Class III malocclusion and anterior open bite managed to achieve an overjet of 2.4 mm and an overbite of 0.5 mm with a CA and optimized attachments with gingival bevels placed on the anterior teeth and on the maxillary arch [19].

#### 4.1.1. Desired Tooth Movements

Tooth movements and forces that are difficult to achieve with CAs include incisor torque control during retraction, extrusion and canine rotation [38]. Rotations and intrusions must be overcorrected at the end of each movement. The anterior open-bite treatment has an increased risk of posterior open bite and increased overbite, since the elastic CAs are prone to horizontal deformation. This can be avoided by diversifying the aligner-induced forces. Planning a mandibular reverse curve of Spee and an accentuated maxillary curve of Spee and using attachments with elastics on the upper and lower premolars were proposed as an answer to these problems [38]. Digital models of tooth movements and plan attachments are divided into stages by software that provide a visual analysis of open-bite treatment [37]. CAs use computer-aided planning of forces that induce tooth movements and support the teeth on their buccal, labial, mesial, distal, lingual and occlusal surfaces.

#### 4.1.2. Posterior Intrusion and/or Anterior Extrusion

The intrusive force exerted on the posterior segments by aligners for the counterclockwise rotation of the mandible is not merely generated by the thickness of Invisalign^®^, which is only 0.76 mm per dental arch. Instead, this intrusive force must be programmed with ClinCheck ^®^ 5.2 [8], by adding posterior occlusal bite-block attachments, but more recent research has shown that they have no impact on the efficacy of open-bite treatment [10]. However, other authors suggest that no planning is required, arguing that the bite block effect created by the thickness of the aligner’s thermoplastic material on posterior teeth is helped by the patient’s biting force, leading to an intrusion of the maxillary mesiobuccal cusps of 0.47 mm and to a bite deepening effect [9].

The anterior extrusion necessary for open-bite treatment requires a strong posterior anchorage. This is achieved by aligner attachments used as retention auxiliaries and force delivery systems. Attachments help control the posterior molar torque. Mini-plates, mini-screws and elastics are recommended for posterior intrusion in cases of excessive gingival display or in skeletal open bite. They improve the molar intrusion caused by the thickness of the thermoplastic aligner materials through the bite block effect and by the occlusal forces of chewing and swallowing. Posterior bite blocks on the occlusal surfaces of molars should be also considered in some patients [9]. The anterior distalization achieved with mini-screws, mini-plates and extractions can be improved by applying power arms for better root control and by using elastics attached on the lower first molars and upper canines. The intermaxillary elastics provide better anchorage and prevent the bowing effect [38]. The attachments can be placed a few weeks after starting wearing the aligner, allowing for gradual adaptation [37]. Alternation of active and inactive distalization periods could be a way of overcoming the disadvantage of the uniform thickness of aligners. The anterior retraction can be planned as canine distalization, incisor intrusion and protraction, which alternates with partial incisor retraction [38].

The treatment planning with ClinCheck^®^ Pro 6.0 aims to achieve posterior intrusion as well since attachments were introduced in 2011 in the Invisalign^®^ G4 enhancements [9]. Some studies used this temporal inclusion criterion for this very reason—the adoption of algorithms for molar intrusion [7].

#### 4.1.3. Refinements and Treatment Adjustments

Refinements involve a new set of aligners for final open-bite correction and esthetic improvement. Aligner refinements are accompanied by optimized attachments and elastics. About 70%−80% of patients treated with Invisalign^®^ necessitate periodic refinements [16]. Out of a total number of 45 patients, no refinements were needed only in 3 patients [9]. Reasons for refinement in open-bite treatment may occur when attachments impact the accuracy of tooth movements. Even if vertical movements were found to differ significantly than anticipated, only the intrusions of incisors were the most inaccurate movements, with incisor extrusion being accurate in a retrospective study on refinement requirements that also included three anterior open-bite cases [28].

#### 4.1.4. Retention Protocol

Retention protocols for open-bite treatment with CAs are those used in fixed appliances [11,29]. Vacuum-formed retainers are usually recommended, as they have a bite-deepening effect like that of aligners, and their occlusal coverage maintains molar vertical position. Hawley-style, Essix and bonded retainers are also used [30,39]. Optimal open-bite retention is achieved when retainers exert higher occlusal forces on the posterior teeth by applying additional layers of material [1]. Maxillary and mandibular aligners are also used for retention, their posterior intrusive force being like posterior bite blocks. Higher retention rates than in fixed appliances may be explained by aligners acting as retainers of the vertical dimension and not as openers of the mandibular plane [11].

### 4.2. Influencing Factors and Treatment Outcomes

Reconciling patients’ esthetic and functional expectations with oral health, stability and the desired cephalometric values requires overcoming a series of issues related to the individual characteristics of patients, the orthodontist’s training and experience and aligner usage protocols and manufacturing limits.

#### 4.2.1. Patient-Related Factors

Aligner treatment particularly relies on patient compliance. As is the case with removable appliances, for aligners to be worn continuously, a patient’s involvement is very important [38]. This is especially true when the treatment period reaches as high as 26 months, requiring the replacement of 125 aligners [37], or even 34 months or 3 years [8,30], in situations in which refinements are needed. Reports of abandonment are scarce due to the inclusion criteria related to non-growing patients, finalized orthodontic treatment or to the retrospective nature of studies, but they ranged from 2.63% to 38.5% [39,40]. This is why the sample size of studies assessing clear aligner efficiency is usually low. Compliance challenges usually involve patients who fail to use the elastics as recommended [38]. Compliance stimulators in the form of lingual attachments for upper- and lower-anterior teeth can improve open-bite treatment outcomes [31]. Missed appointments and prolonged aligner wear times were also cited as reasons for poor patient compliance [21].

Patients tend to refuse the extraction of the first premolars or the surgically assisted palatal expansion [31,37], which is why extractions are usually absent in clear aligner patients. Crowding is managed with interproximal reduction and arch expansion [7].

In patients with poor periodontal support caused by gingival recession, it is important to minimize periodontal tissue stress and forces on the adjacent teeth by reducing the range of tooth movement and by controlling root movement so that the roots do not extend beyond the margin of the alveolar crest [41]. However, predicting root movements with CAs can be challenging, as digital planning focuses on images of tooth crown movements [42]. Periodontal issues, such as gingival recession and dental migration, can be improved with CAs. CAs preserve periodontal health due to improved plaque control and hygiene, minimal soft-tissue injuries and limited impact on tooth mobility [43]. By adding high-frequency vibration (HFV) to clear aligner treatment, increased bone density with new bone formation labially to the lower incisors was achieved in one open-bite patient who used an HFV of 120 Hz for 5 min per day, as shown by cone-beam CT [27]. A patient with anterior open bite on second incisors and slight gingival recession received corticotomy-assisted treatment with CA. The piezocision corticotomy was performed distally and mesially to second incisors and canines to accelerate open-bite treatment and improve root movement. The extrusion and torque were optimized with an attachment added to the second incisors [44].

Bruxism has little impact on clear-aligner open-bite treatment. Aligners could act similarly to night guards or soft bite plates used for bruxism treatment, but CAs strain jaws by increasing the activity of masticatory muscles, leading to temporary mild jaw tenderness [45] and have no significant effect on sleep bruxism [46]. Clear aligner treatment itself triggers episodes of awake bruxism, as shown by the increased masticatory muscle activity [47]. Daytime teeth grinding associated with a soft-bite-plate use leads to mandibular rotation. However, no statistically significant mandibular autorotation was found in patients treated with Invisalign^®^ for anterior open-bite malocclusion for a mean period of 20 months [12].

In patients previously treated with fixed orthodontic appliances for amelogenesis im-perfecta, aligner use is recommended, as retreatment with braces is not advised [32]. So far, there are no reports using the lack of previous orthodontic treatment as an inclusion criterion. Previous treatment with fixed orthodontic appliances is usually a reason for a patient’s choice for CA, as a means of not repeating an unpleasant experience [20], but it may also be the cause of poor compliance [48]. Previous orthodontic treatment with fixed appliances may also explain current abnormal occlusion, due to incomplete treatment [31] or relapse [49].

Regarding patients with esthetic demands, CAs may correct open bite in hyperdivergent facial types associated with a distal movement of upper molars of up to 2–3 mm, due to good control of the vertical dimension in upper molars and effective incisor torque for maxillary molar distalization [50]. Good control of molar extrusion is required in patients with hyperdivergent skeletal pattern and high mandibular plane angle (MP-SN > 38°) to prevent the backward rotation of the mandible [13]. Open bite associated with facial hyperdivergence is characterized by maxillary excess, with an excessive incisor extrusion to compensate for the existing skeletal deficit. Upper incisor extrusion is considered unstable [12] and may cause further excessive gingival display, which is why the extrusion of incisors should not exceed 1–2 mm [22,33].

Table 6 has a concise summary of the clinical recommendations based on patient-related factors in clear aligner treatment.

#### 4.2.2. Clinician-Related Factors

Aligners demand complex initial treatment planning, as subsequent corrections are impossible to make at each visit. Orthodontists should have good knowledge of the characteristics of aligners and attachments, tooth movement biomechanics and the limitations of each case. Shorter dental chair treatment means less inconvenience for the patient, but more effort for the dentist. The orthodontist prioritizes and selects tooth movements and attachments based on suggestions made by the planning software [10]. Aligner efficiency was evaluated in some cases only in experienced orthodontists with an annual minimum of one hundred Invisalign^®^ treatments [8]. In a study on 232 orthodontists, including clear aligner providers, all practitioners were aged over 45 years, with more than 10 years’ experience [15]. Open-bite cases consecutively treated by the same Invisalign^®^ provider were also of interest, possibly on account of the homogeneity of materials and methods [7,9,23]. However, this could be a source of bias, as treatment outcomes may be strongly influenced by the orthodontist’s unique working style [7].

Tongue posture reeducation through myofunctional therapy averts the risk of open-bite relapse [50], which is a decrease in overbite higher than 0.3 mm than in the absence of tongue positioning issues [11]. Voluntary clenching of teeth and hard chewing gum may be used to improve the vertical impact on posterior teeth and to increase the strength of masticatory muscles, the occlusal contact area and the occlusal sagittal relationship, especially in hyperdivergent patients with anterior open bite [33]. Table 7 provides a concise summary of the clinical recommendations based on clinician-related factors in clear aligner treatment.

#### 4.2.3. Technology-Related Factors

The tooth movements that are the most difficult to achieve with aligners should be analyzed considering attachment use [19], with mini-screws and mini-implants [30], and differences among types of teeth and between maxillary and mandibular positioning. There is also a tendency toward upward tooth movement caused by the additional forces created by changes in the fixation system of aligner attachments due to the tipping of teeth. Moreover, aligner thickness must reconcile comfort with increased force requirements [51]. Even if various brands are also targeting open-bite treatment (AirNivol, ALL IN, Dental Stealth, Effect Aligners, F22, Orthocaps, Smart Evolution, Smile Clear or Smiletech [52]), Invisalign^®^ (Align Technology, Inc., San Jose, CA, USA) was the most frequently used aligner. Only one case report has used the Nuvola^®^ OP System, which combines aligners with a myofunctional elastodontic device [33].

Considering that Invisalign^®^ CAs provide ongoing improvements to the SmartTrack material for improved fit of the trays, attachments and software quality [53], regularly updated reviews on their performance are required.

Attachment use has considerably improved the anterior extrusion, even if this was considered in a 2015 review of aligner orthodontic efficiency as the least accurate tooth movement, with only 18% accuracy of extrusion in the upper central incisors and 25% in the lower ones [2]. Before the 2011 improvements, reviews have already noted the difficulties of drawing conclusions given that CAs evolve continuously [40].

### 4.3. Outcome Measures and Comparative Effectiveness

Comparative studies have shown that the treatment success rate was similar in clear aligner and fixed-appliance patients [7,15], but the digital planning programmed tooth movement is overestimated in anterior open-bite patients [10,19].

The use of CAs leads to anterior extrusion in the absence of auxiliary vertical anchorage in the posterior teeth. A significant incisor extrusion of 1.5 mm on average achieved in patients with an average initial open bite of −1.1 mm suggests that open-bite closure may be due almost entirely to the anterior extrusion, with minimal changes in the vertical molar movement and in the mandibular plane angle [8,24]. Several studies reported a statistically significant intrusion in mandibular molars and only a minor intrusion in the maxillary molars [8,11] but a statistically significant intrusion in both maxillary and mandibular molars [9] or a statistically significant intrusion in the maxillary molars and a non-statistically significant intrusion in the mandibular molars [12] were also found. When the incisors are significantly retracted, the anterior open-bite closure may be attributed to the relative extrusion of incisors through retroclination instead of absolute incisor extrusion [11,23]. Interproximal reduction and arch expansion can be used to manage crowding and to retrocline the anterior teeth [11].

CAs have the advantage of lacking the extrusive effect on molars seen with fixed orthodontic appliances that might aggravate the anterior open bite [19]. Dental movements triggered by the occlusal forces of CAs covering the masticatory surfaces of teeth do not impair the stability of anterior overbite as the counterclockwise mandibular rotation achieved by brackets does [37]. Moreover, aligner molar intrusion is achieved simultaneously with anterior extrusion without requiring any transpalatal or facial arches, as the thickness of aligner thermoplastic exerts enough intrusion on posterior teeth [54,55]. The teeth not intended for movement stay anchored in place [28].

The downside of aligner open-bite treatment is the risk of posterior open bite [12], insufficient tooth movement, with lower extrusion and higher intrusion [56], caused by weak orthodontic forces, inadequate fastening, lack of specific force-bearing points and the opposing force of dental crowns. The extrusion accuracy provided by CAs amounts to considerably less than 50%, with reports of just 29.6% [39] and 30% [2], the lower canine being the most difficult to move [39]. Even in cases where, in fact, an intrusion was planned, only 26% of the predicted intrusion for lower incisors and 51% for canines was achieved [53].

However, data published in 2009 [39], 2015 [2], and 2018 [54], respectively, are increasingly challenged by more recent Invisalign^®^ improvements. A review of various tooth movements generated by aligners alone that was published in 2017 has shown that the anterior extrusion was predictable up to 2.5 mm, moderate within the range of 2.5–3 mm and difficult when exceeding 3 mm, whereas the posterior intrusion was predictable up to 0.5 mm, moderate between 0.5 and 1 mm and difficult above 1 mm [57]. The extrusion of maxillary incisors remains problematic, but it can be improved by using attachments, by phasing treatment and by diversifying the ways in which forces are exerted to alternate distalization periods with extrusion intervals and to achieve supplemental movements such as retraction or retroclination [38].

Post-treatment follow-up by superimposition of lateral cephalograms should indicate the counterclockwise rotation of the mandibular body [58]. The risk of bias during superimposition assessment in adult patients treated for anterior open bite performed by graduate students and orthodontic practitioners showed no significant superimposition bias, but the raters had a slight tendency to report no change or a closure in the mandibular plane angle when they were told that the treatment was performed with CAs [59].

### 4.4. Recommendations for Improved Aligner Use

CA efficiency in open-bite correction may be improved by creating small interdental spaces prior to treatment. Moderate overcorrection can be used to manage the unpredictable rotations of upper and lower canines [39].

CA treatment can be associated with fixed appliances [15]. A hybrid approach was successfully performed in a pediatric patient with mixed dentition and anterior open bite, when CAs were used simultaneously with fixed partial lingual appliances after orthopedic facemask therapy [60]. CAs can also be used in open-bite malocclusions as a second-phase treatment in interventions initiated with a Carriere Distalizer correction appliance [34].

Performing a twice-weekly aligner change may help open-bite closure by increasing the time available for the prescribed vertical movements, with 0.49 mm more bite closure on average [10]. The use of optimized extrusion attachments on incisors may compensate for this treatment prolongation by shortening treatment duration [23].

The bite closure overestimation provided by the digital planning software can be managed by increasing the prescribed tooth movements and by using reinforced vertical anchorage in posterior teeth [10].

Virtual articulators can be used to create and improve a functional occlusion, leading to higher accuracy of clear-aligner open-bite treatment planning [35].

### 4.5. Study Limitation

The present review has several limitations. Primarily, it focused on studies involving Invisalign^®^ (Align Technology, San Jose, CA, USA) and its associated ClinCheck^®^ Pro 6.0 software. However, there are numerous other clear aligner systems in clinical use, including ClearCorrect^®^ (Institut Straumann AG, Basel, Switzerland) with ClearPilot 10.0 software; Reveal^®^ (Henry Schein, Melville, NY, USA); SureSmile^®^ (Dentsply Sirona, York, PA, USA), with Open Software and Digital Lab; Clarity Aligner Flex and Force^®^ (3M ESPE, Maplewood, MN, USA); and Spark^®^ (Ormco Corp, Envista, Brea, CA, USA), with Approver R15.6 software, among others [14]. As such, the brand-specific focus may limit the generalizability of our findings, particularly given the potential differences in aligner materials, treatment mechanics and digital workflow algorithms. Future research comparing the efficacy of different clear aligner systems is warranted to enhance the external validity of conclusions regarding anterior open-bite correction.

Most included studies were observational retrospective studies and case reports/series, which inherently present risks of selection bias, limited control of confounding variables and reduced generalizability. Additionally, studies were often conducted in individual or private clinical settings, possibly reflecting treatment protocols tailored to specific patient populations or practitioner preferences.

Importantly, the lack of long-term follow-up data across most studies restricts conclusions regarding the stability of anterior open-bite correction with clear aligners. Given the high relapse risk associated with this malocclusion, future studies should prioritize extended follow-up periods to evaluate treatment durability.

Although gray literature sources such as OpenGrey and Google Scholar were searched, they yielded no additional eligible studies. This may indicate a limited body of unpublished or non-indexed research on this topic, thereby introducing a risk of publication bias, as studies with negative or inconclusive outcomes may remain unreported.

Furthermore, while anterior open-bite treatment is relevant across age groups, the included studies predominantly evaluated adult populations. Differences in skeletal maturity, growth potential, compliance and treatment response between adults and younger patients may influence outcomes. Therefore, future research should consider comparative studies across age groups to improve understanding of age-related variability in clear aligner efficacy.

Lastly, to advance clinical applicability, future multicenter prospective trials, including comparisons among various aligner systems, are needed. These should also assess the predictability of digital treatment planning simulations and the long-term stability of results to establish evidence-based recommendations for clear aligner use in anterior open-bite management.

## 5. Conclusions

This scoping review explores the current evidence on the use of clear aligners, particularly Invisalign^®^, in the management of anterior open bite in adults. The available literature suggests that clear aligners may offer certain advantages, including improved esthetics, better periodontal outcomes and the benefits of digital treatment planning. However, their effectiveness in complex malocclusions appears limited, often necessitating the use of adjunctive approaches such as mini-screws and elastics to improve treatment predictability. The success of aligner therapy appears closely linked to patient compliance and the accuracy of digital simulations, which may not always reflect actual clinical outcomes. While other systems such as Spark^®^, ClearCorrect^®^ and SureSmile^®^ are emerging, limited peer-reviewed data are currently available on their efficacy in treating anterior open bite. This represents both a limitation of the current review and an important avenue for future research. Given the predominance of observational and retrospective studies in the existing evidence base, further high-quality, comparative research—particularly randomized controlled trials and long-term outcome studies—is warranted to better define the role of clear aligners in anterior open-bite correction.

## Figures and Tables

**Table 1:** Key biomechanical considerations and influencing factors in open-bite correction with clear aligners.

Topic	Supporting References			
	Observational Studies		Case Report/Series Studies	
	Retrospective Studies (13 Studies)	Prospective Cohort Studies (1 Studies)	Case Report Studies (13 Studies)	Case Series Studies (3 Studies)
Posterior intrusion	[7,8,9,10,11,12,13,14]	[15]	[16,17,18,19]	[20]
Anterior extrusion	[7,9,10,11,12,13,14,21,22,23,24,25]		[19,26,27]	
Refinements and treatment adjustments	[9]		[16]	
Retention protocol	[11]		[28,29,30]	
Patient-related factors	[8,12,13,21,22]	[15]	[27,30,31,32,33]	[20,32]
Clinician-related factors	[7,8,9,11,23]	[15]	[33]	
Technology-related factors	[10]		[19,30,33,34]	[35]

**Table 2:** Factors influencing treatment pertaining to patient characteristics.

Patient-Related Factors	Effects and Action	Reference(s)
Failure to use the aligners correctly or continuously	Lingual attachments for upper- and lower-anterior teeth	[8,21,30,31]
Periodontal disease	High-frequency vibration and corticotomy in addition to clear aligner treatment	[27]
Bruxism	Awake bruxism during clear aligner treatment has no significant impact on outcomes	[12]
Previous treatment with fixed appliances	A possible cause of open bites and a reason for patients’ choice of CAs or poor compliance	[20,31,32]
Smile and facial appearance	In hyperdivergent facial types, upper-incisor extrusion should not exceed 1–2 mm due to the risk of excessive gingival display	[13,22,33]

**Table 3:** Factors influencing treatment with reference to orthodontists.

Orthodontist-Related Factors	Effects and Action	Reference(s)
Pre-treatment planning	Skills and willingness to use computer-aided planning	[10]
Training and level of experience	An annual minimum of 100 clear aligner treatments Orthodontist’s unique working style → uniform materials and methods and yet a source of research bias	[7,8,9,15,23]
Post-treatment follow-up	Tongue posture reeducation and tooth clenching	[11,33]

**Table 4:** Summary of clinical observational studies evaluating clear aligner efficacy in the correction of anterior open bite.

	Study Design	Treatment Period	Study Objective	Participants Sample Size	Mechanism of Anterior Open-bite Correction	Clear Aligner Efficacy
1	Garnett et al., 2019 [7], comparative retrospective study	Fixed Appliance Group: 2008–2014; Clear Aligner Group: 2011–2014	To compare fixed appliances and clear-aligner therapy in correcting anterior open bite and in controlling the vertical dimension in adult patients with hyperdivergent skeletal patterns.	17 fixed-appliance patients and 36 clear-aligner patients.	Retro inclination of the upper and lower incisors while maintaining the vertical position of the upper and lower molars.	The clear aligner group showed a slightly greater amount of lower incisor extrusion (*p* = 0.009). Mean overbite changes of 2.28 ± 1.55 mm, not significantly higher than in the fixed-appliance group.
2	Moshiri et al., 2017 [8], retrospective study	18 months	The purpose of this study was to evaluate, by means of cephalometric appraisal, the vertical effects of non-extraction treatment of adult anterior open bite with CA.	Lateral cephalograms of 30 adult patients with a Class I or II open bite.	Bite closure was mainly achieved by a combination of counterclockwise rotation of the mandibular plane, lower molar intrusion and lower incisor extrusion.	An average overbite change of 3.4 ± 1.4 mm.
3	Harris et al., 2020 [9], single-center retrospective study	-	To evaluate the dental and skeletal effects that occur in the correction of anterior open bite with CA.	45 adult patients with an average open bite of −1.21 ± 1.15 mm.	Anterior retraction and extrusion and posterior molar intrusion, with slight mandibular autorotation.	An average overbite change of 3.27 ± 1.09 mm.
4	Blundell et al., 2023 [10], multicenter retrospective study	-	The objective of this study was to investigate and determine the accuracy of Invisalign^®^ treatment in correcting anterior open bite by comparing the predicted outcome from ClinCheck^®^ Pro 6.0 to the achieved outcome for the initial aligner sequence.	76 adult patients with a mean overbite of −1.48 mm ± 1.21 mm.	Anterior extrusion	An average of 66.7% of the programmed ClinCheck^®^ Pro 6.0 open-bite closure.
5	Steele B.P. et al. (2022) [22], retrospective cohort study		To compare the dentoskeletal effects and treatment outcomes of clear aligner therapy versus miniplate-supported posterior intrusion (MSPI) combined with fixed appliances in adult patients with anterior open bite.	Invisalign group: 29 patients; MSPI group: 24 patients.	Anterior extrusion	MSPI resulted in significantly greater maxillary molar intrusion (1.5 mm) compared to Invisalign.
6	Suh et al., 2023 [11], retrospective cohort study	-	This study aimed to examine the stability of anterior open-bite (AOB) treatment with CA.	52 adult patients with an initial overbite of less than −0.5 mm, evaluated at least 1-year posttreatment.	Anterior extrusion, with minor maxillary molar intrusion leading to mandibular counterclockwise rotation.	A mean retention period of 2.1 ± 1.1 years with a mean change in overbite of 3.3 ± 1.5 mm and 94% stability.
7	Pokorna et al., 2022 [12], retrospective study	20 months	The study aimed to assess the cephalometric changes in patients with a clinically proven anterior open bite after clear aligner treatment (Invisalign^®^). The amount of planned movement was also compared with the amount of movement that actually occurred, measured as 8 on the radiographs.	30 adult patients with a bite depth of <0 mm.	Intrusion of the lateral segments and anterior extrusion.	A mean bite deepening of 2.71 mm. Anterior extrusion of 0.95 to 1.06 mm in the maxilla and 0.38 mm in the mandible. Molar intrusion occurred in the maxilla (0.66 to 0.83 mm) during treatment.
8	Todoki et al., 2020 [15], observational prospective cohort study	30 months	This study reports on the overall success rate of anterior open-bite orthodontic treatment in the adult population across the United States, as well as 4 major treatment modalities and other factors that may influence treatment success.	29 adult patients with a mean open bite of −2.35 mm in the total number of 254 orthodontic patients.	Posterior intrusion	Similar success rates of 81% for aligner and fixed-appliance treatment.
9	Burashed et al., 2023 [23], retrospective cohort study	-	The aim of this retrospective study was to compare the efficacy of anterior open-bite correction with Invisalign^®^ when using optimized extrusion versus conventional attachments.	86 adult patients with an overbite of ≤0 mm on all 4 incisors.	Conventional horizontal and rectangular attachments or optimized extrusion attachments on incisors.	An efficacy of anterior open-bite correction of 58.4%, with similar results in patients with conventional versus optimized attachments, the latter having a shorter treatment duration.
10	Khosravi et al., 2017 [24], retrospective study	-	To assess the nature of overbite changes with the Invisalign^®^ appliance. The study sample included 68 patients with normal overbites, 40 with deep bites and 12 with open bites.	12 adult patients with mild to moderate open bites.	Anterior extrusion	A median deepening of overbite of 1.5 mm ± 0.5 mm.
11	Kau et al., 2017 [14], retrospective study	-	A comparison of pre-treatment and posttreatment scores was conducted using the PAR index and ICON.	Invisalign^®^ 2 groups: Anterior open-bite patients: 23; Control group: 77.	Posterior intrusion and anterior extrusion.	Invisalign is effective in correcting anterior open bites, achieving outcomes comparable to non-open-bite patients when well-planned and well-executed.
12	Chamberland & Nataf, 2024 [25], retrospective comparative study		This study aimed to compare the treatment effect and mechanisms of open-bite closure between patients treated with braces and TADs double-arch intrusion and those treated with CAs.	The TAD group included 18 consecutively treated patients from the main author. The CAT group consisted of 16 selected patients from three different orthodontists.	Anterior extrusion	A significant reduction in vertical dimensions temporary only in skeletal device treatment, whereas clear aligner treatment showed a significant extrusion of 1.22 ± 0.42 mm of the lower incisors.
13	Rask K et al., 2021 [13], retrospective study	-	The purpose of this study was to compare changes promoted by CAs and traditional fixed appliances in cephalometric measurements of the vertical dimension and molar position in adult patients with Class I malocclusion treated with non-extraction.	Pre- and post-treatment lateral cephalometric radiographs of adult patients treated with either CAs (*n* = 44) or traditional fixed appliances (*n* = 22).	Posterior intrusion and anterior extrusion.	Clear aligners can effectively control the vertical dimension and aid in anterior open-bite closure in adults, potentially better than fixed appliances in non-extraction protocols.
14	Karalikkattil, T.L. et al., 2024 [21], retrospective cohort study	18 months	This study aims to contribute to the existing body of knowledge by evaluating the effectiveness of Invisalign^®^ treatment on open-bite correction through a retrospective analysis of patient records.	A retrospective analysis was conducted on a cohort of 50 patients with open bites who underwent Invisalign treatment.	Anterior extrusion	Treatment evaluation showed that the mean overbite improved to +1.5 mm (SD = 0.8).

**Table 5:** Invisalign^®^-based studies on antersior open-bite treatment.

Study	Year	Study Design and Invisalign^®^ Usage
Garnett et al. [7]	2019	Retrospective Comparative—compared Invisalign^®^ vs. fixed appliances
Moshiri et al. [8]	2017	Observational Retrospective—cephalometric evaluation of Invisalign^®^
Harris et al. [9]	2020	Retrospective—evaluated open-bite closure using Invisalign^®^
Waxler et al. [17]	2021	Case Report—skeletal open-bite corrected with Invisalign^®^ + TADs
Vadera et al. [19]	2023	Case Report—skeletal Class III malocclusion + dentoalveolar anterior open bite using CA
Blundell et al. [10]	2023	Retrospective—predictability of anterior open bite with Invisalign^®^
Giancotti et al. [20]	2017	Case Series—Invisalign^®^ used in open-bite cases
Schupp et al. [29]	2010	Case Series—focused on Invisalign^®^ system
Suh et al. [11]	2023	Observational Longitudinal—studied short-term stability of Invisalign^®^ treatment
Karalikkattil al. [21]	2024	Retrospective—Invisalign^®^ effectiveness for anterior open bite
Steele et al. [22]	2022	Retrospective—clinical dentoskeletal effects and treatment outcomes of Invisalign^®^ patients versus miniplate-supported posterior intrusion (MSPI) combined with fixed appliances
Burashed et al. [23]	2023	Comparative Retrospective—Invisalign^®^ optimized vs. conventional attachments
Khosravi et al. [24]	2017	Retrospective—overbite management with Invisalign^®^
Rodriguez et al. [34]	2012	Case Report—non-extraction open-bite treatment with Invisalign^®^
Haubrich et al. [35]	2023	Case Series—follow-up on aligner open-bite treatments
Kau et al. [14]	2017	Retrospective—Invisalign^®^ effectiveness for anterior open bite

**Table 6:** Clinical recommendations based on patient-related factors in clear aligner treatment.

Recommendation	Details
Improve Patient Compliance Including Long Term Treatments	- Ensure clear communication about wearing aligners 20–22 h daily; - Schedule regular follow-ups and use digital reminders; - Use compliance stimulators (e.g., lingual attachments) in complex cases.
Address Orthodontic Extractions and Surgical Concerns	- Consider alternatives like interproximal reduction (IPR) or arch expansion for patients refusing extractions; - Provide detailed counseling on necessary surgical procedures.
Monitor Periodontal Health	- Evaluate periodontal health before treatment, especially with gingival recession; - Limit tooth movement in patients with compromised periodontal support; - Consider using high-frequency vibration (HFV) and corticotomy-assisted treatments for better bone density.
Manage Bruxism and Jaw Tension	- Monitor bruxism and adjust aligner designs to minimize discomfort; - Educate patients on potential jaw tenderness and provide pain relief strategies.
Consider Previous Orthodontic Treatment	- Assess previous orthodontic relapse or unfinished treatment before aligner therapy; - Set realistic expectations for patients with prior fixed-appliance treatments.
Meet Esthetic Demands in Complex Cases	- Carefully control vertical dimension and incisor torque, especially for hyperdivergent patients; - Monitor and limit molar extrusion to prevent skeletal shifts; - Consider molar distalization for open-bite correction when needed.
Enhance Esthetic and Functional Outcomes	- Use strategically placed attachments for better tooth movement efficiency in complex cases; - Tailor the treatment plan to the patient’s individual esthetic and functional needs.

**Table 7:** Clinical recommendations based on clinician-related factors in clear aligner treatment.

Category	Clinical Recommendations
Comprehensive Treatment Planning	- Develop a detailed and precise initial plan, since mid-course corrections are not possible; - Use the brand software to prioritize tooth movements and attachments.
Clinician Expertise and Experience	- Orthodontists should have strong knowledge of aligner biomechanics, attachments and limitations; - Optimal results are seen with at least 10 years of experience or 100+ cases per year.
Case Selection & Bias Considerations	- Be aware that treatment success may be influenced by the orthodontist’s unique working style; - Research studies should control for bias when evaluating aligner effectiveness.
Managing Open-Bite Cases	- Myofunctional therapy helps reeducate tongue posture and prevents open-bite relapse; - Tongue positioning issues can lead to a decrease in overbite (>0.3 mm); - Hard gum chewing and voluntary clenching help improve vertical control, occlusal contact and muscle strength in hyperdivergent patients.
Patient-Centered Approach	- Educate patients on aligner compliance and the importance of auxiliary interventions; - Consider skeletal patterns (e.g., hyperdivergence) when planning treatment; - While aligners reduce chair-side time, they require greater precision and effort from the orthodontist.
